# Topical 15% trichloroacetic acid versus silver nitrate cauterization in the management of idiopathic childhood epistaxis: a prospective randomized clinical trial

**DOI:** 10.1007/s00431-026-07483-4

**Published:** 2026-11-12

**Authors:** Ibrahim Gehad, Saad Elzayat, Youssef Zaki, Salma Elmetwally Kandil, Ahmed S. Abdelhamid

**Affiliations:** 1 Department of Otorhinolaryngology-Head and Neck Surgery, Faculty of Medicine, Kafrelsheikh University, El-Geish Street, Kafrelsheikh, 33155 Egypt; 2 Anesthesiology, Department of Surgical Intensive Care and Pain Medicine, Faculty of Medicine, Tanta University, Tanta, Egypt

**Keywords:** Epistaxis, Chemical cauterization, Silver nitrate, TCA, Idiopathic, Pediatric

## Abstract

The purpose of the study is to compare the outcomes of trichloroacetic acid (TCA) to silver nitrate chemical cauterization in managing pediatric epistaxis. In this prospective randomized assessor-blinded clinical trial, of 172 randomized patients, 157 pediatric patients with idiopathic anterior epistaxis were divided into 2 groups in a ratio 1:1, where group (A) was treated with silver nitrate 25% after local anesthesia application and group (B) was treated with 15% TCA locally applied to the bleeder. Patients were assessed upon recurrence, pain, and crusting at 1st, 2nd, 4th, and 12th week post-intervention. The incidence of epistaxis was not significantly different between the two groups. Pain was significantly greater in the TCA group (*P* = 0.008*). Nasal crusting was slightly greater in the TCA group, but the difference was not statistically significant.

*Conclusion*: Although not widely utilized in the management of pediatric epistaxis, TCA is considered an effective chemical cautery agent for the management of childhood epistaxis, with results comparable to those of silver nitrate in terms of recurrence but was slightly worse in terms of pain after intervention.

*Trial Registration*: Number NCT06836791, Date of registration 02/19/2025.

**Table:** 

**What is Known:** • *Epistaxis is a very distressing complaint in pediatric otorhinolaryngology.* • *One of the utilized modalities nowadays that proved efficacy in the management of epistaxis is the chemical cauterization of the prominent vessels in Little’s area.*	
**What is New:** • *This is the first study to compare the efficacy of silver nitrate to TCA in epistaxis treatment.* • *TCA is considered an effective chemical cautery agent for the management of childhood epistaxis, with results comparable to those of silver nitrate agent.*

**What is Known:**

• *Epistaxis is a very distressing complaint in pediatric otorhinolaryngology.*

• *One of the utilized modalities nowadays that proved efficacy in the management of epistaxis is the chemical cauterization of the prominent vessels in Little’s area.*

**What is New:**

• *This is the first study to compare the efficacy of silver nitrate to TCA in epistaxis treatment.*

• *TCA is considered an effective chemical cautery agent for the management of childhood epistaxis, with results comparable to those of silver nitrate agent.*

## Introduction

Epistaxis is a very distressing complaint in otorhinolaryngology that affects nearly 10–12% of the population worldwide [1]. Epistaxis in children is even more common. About half of the children had one episode or more of epistaxis by the age of 10 [2].

The source of epistaxis may be anterior or posterior; in children, it is commonly anterior originating mainly from the anterior part of the nasal septum called Little’s area in which a confluence of veins named Kiesselbach’s plexus is the main to blame [3]. Anterior epistaxis is commonly related to local trauma such as nose picking or crust formation or local inflammation such as upper airway infection and vestibulitis due to bacterial inhabitants that lead to crusting and precipitation of epistaxis episode [3].

In the literature, there are various modalities for the treatment of anterior epistaxis such as nose pinching and anterior nasal packing in emergent cases and medical treatment in between attacks which are considered first aid and whenever failed, we shift to alternative methods which are considered invasive but more effective [4].

One of the utilized modalities nowadays that proved their efficacy in the management of epistaxis is the chemical cauterization of the prominent vessels in Little’s area [5]. In 1932, Little was the first to introduce the idea of using silver nitrate as a chemical cautery agent in the treatment of anterior epistaxis that results in coagulation of bleeding vessels and tissue destruction [6].

Another option for chemical cauterization is trichloroacetic acid (TCA) which causes protein denaturation [7]. Trichloroacetic acid is available, inexpensive, and easily used as a chemical cautery solution. However, there is no adequate evidence in the literature for using TCA in the treatment of epistaxis yet.

Therefore, we aimed this study to explore the efficacy of trichloroacetic acid as a chemical cauterization agent and compare it to silver nitrate in the treatment of idiopathic epistaxis in children.

## Patients and methods

### Study design and population

This was a prospective randomized assessor-blinded study that was held at our tertiary referral center, Otorhinolaryngology. The trial was registered retrospectively at ClinicalTrials.gov (NCT06836791) on 20 February 2025. A total of 184 consecutive pediatric patients were assessed for eligibility between March 2023 and January 2026. Twelve patients were excluded before randomization: nine did not meet the eligibility criteria and three declined participation. The remaining 172 patients were randomized equally into two groups (86 patients per group). The institutional ethical committee of the institute approved the study according to the Helsinki Declaration of 1996 with approval No. (KFSIRB200).

All patients’ parents or assigned caregivers willingly signed a written informed consent for participation in this trial. Patients who sought help complaining of recurrent epistaxis episodes either with or without active bleeding at the time of presentation were offered to participate in the study. All patients aged between 4 and 16 years with at least once weekly in the last 4 weeks with visible anterior bleeding source either unilateral or bilateral from Little’s area were included in the study.

**Fig. 1:**
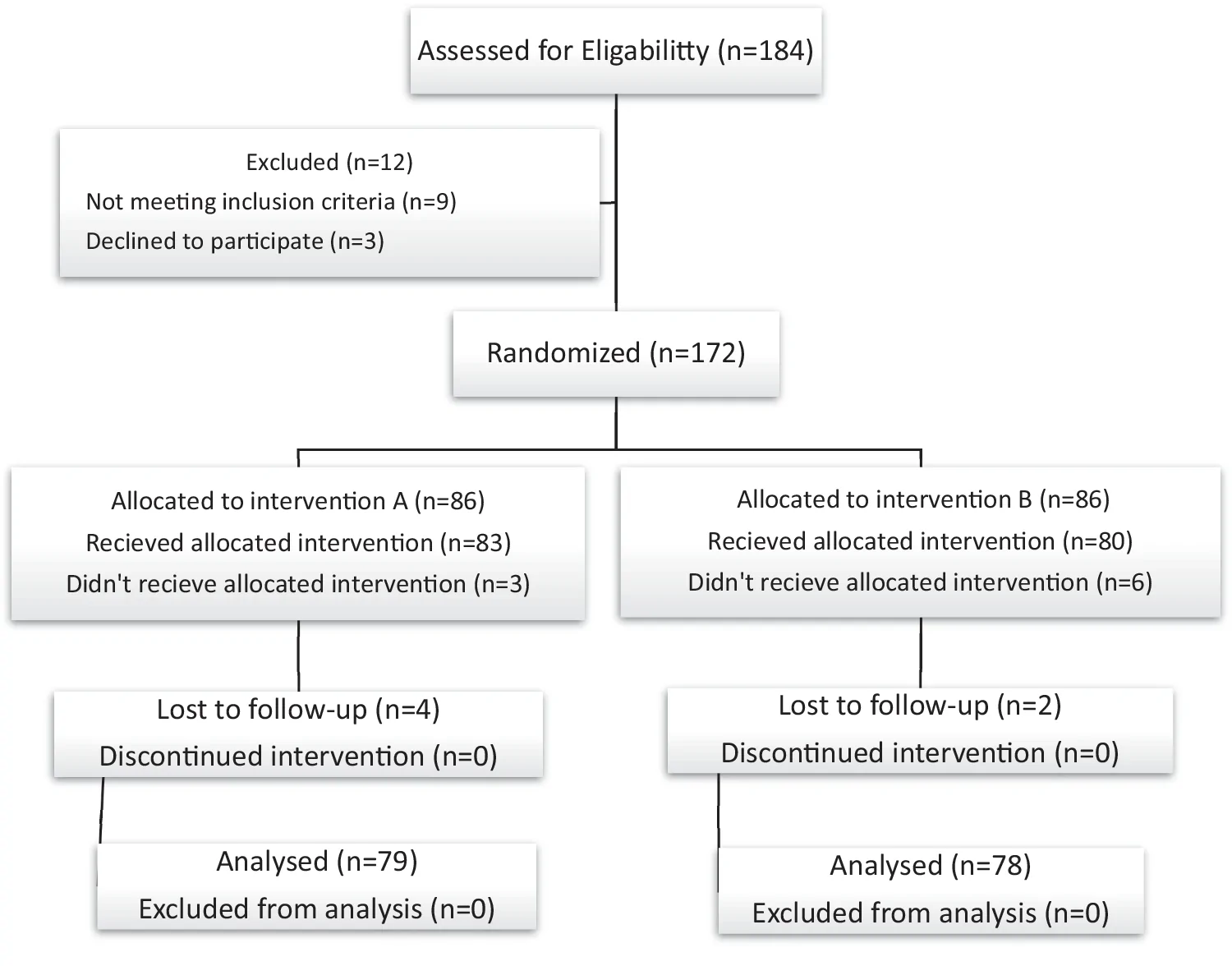
Flowchart showing the methodology of the clinical trial

All patients with documented coagulopathy, those with sinonasal or nasopharyngeal masses, with active rhinosinusitis or severe allergic rhinitis or severe septal deviation, with recent nasal trauma in the last month, history of previous nasal cauterization, those who did not show localized bleeder on examination or with history of allergy to our treatment or local anesthesia were excluded. Patients who did not show up for their follow-up visits were also excluded as mentioned in flow chart (Fig. 1).

For the purposes of this study, idiopathic epistaxis was defined as recurrent anterior epistaxis in the absence of an identifiable local or systemic underlying cause following clinical assessment. Thus, idiopathic epistaxis was considered a diagnosis of exclusion.

All patients underwent complete history taking; their parents filled out a questionnaire for exclusion criteria, and a thorough general and nasal examination was conducted with special attention to locating the bleeder. Complete blood count and coagulation profile were done as a routine checkup for all participants prior to chemical cauterization for epistaxis.

###  Sample size calculation

The sample size was calculated based on the incidence of postoperative pain within anterior nasal packing and silver nitrate cautery groups, respectively [8], and that group 1 to 2 ratio will be 1:1; the sample size is calculated using OpenEPI software to be 78 within each group; 10% of total sample will be added to compensate for potential drop out so each group will include 86 patients at power of study 80% and confidence level 95%.

### Blinding and randomization

Randomization was performed using a computer-generated block randomization sequence with a fixed block size of four. The sequence was generated by one of the investigators. Allocation was concealed using sequentially numbered, opaque, sealed envelopes—handed by a separate nurse—which were opened sequentially at the time of patient assignment. All the interventions were done by the same principal investigator. The follow-up was done in separate settings by two independent investigators who were blinded to treatment allocation and had no access to the randomization information. Allocated patients of the same group were given different schedules to avoid their interaction.

### Interventions

All procedures were performed as office-based procedures under the influence of local anesthesia.

Patients were divided into two groups:

- Group (A)—silver nitrate group: Patients were treated with silver nitrate chemical cauterization; prior to cauterization, 2 cotton packs soaked in 10% lidocaine spray were introduced one into each nostril for 5–10 min. Then, using a cotton-tipped applicator dipped in 25% silver nitrate solution, it was applied to the bleeding point and firmly pressed for 1 min. In case bleeding is noticed, a second application of silver nitrate solution to the area is then made using a cotton tip applicator for 30 s with cauterization of all feeding blood vessels around the bleeding point. This was followed by prescribing an antiseptic soothing cream topically twice daily for 2 weeks.

- Group (B)—the TCA group: Patients underwent chemical cauterization with 15% TCA solution using the same technique.

In both groups, in case there was active nasal bleeding, prior to cauterization, a cotton pack soaked in nasal decongestant oxymetazoline (0.1%) and lignocaine 10% spray was introduced into the nostril for 5–10 min.

Patients were offered topical antiseptic for 2 weeks. Both groups were offered analgesics according to the condition.

### Outcomes

Patients were followed on regular visits at 1 st, 2nd, 4th, and 12th week intervals.

The main outcomes post-chemical cauterization were rebleeding from the nose if present and associated pain 3 days after the procedure. The successful post-chemical cauterization outcome was assessed through complete cessation of epistaxis. Patients with reported recurrence of bleeding from the nose within 12 weeks of intervention were considered failure.

**Fig. 2:**
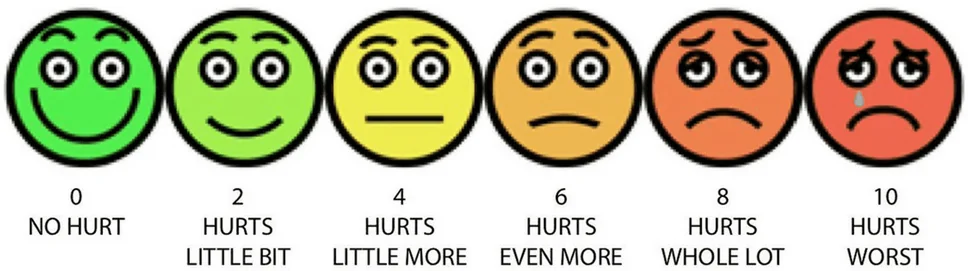
Showing Wong-Baker FACES® Pain Rating Scale

Pain was assessed using the Wong–Baker FACES® Pain Rating Scale [9] represented by a series of faces from 0 (smiling face) to 10 (crying face), with 10 referring to the most intense pain (Fig. 2). Following a brief explanation, the child could select the face that best represents. On the third day after intervention, each child was shown the Wong–Baker FACES® Pain Rating Scale and asked to select the face that best represented their pain. The selected score was recorded by the parent and subsequently reported at the first-week follow-up visit.

The formation of nasal crusts within the two groups was noticed during follow-up. Other complications like septal perforation or rhinitis were also recorded.

### Statistical analysis

Data analysis was performed using the software SPSS (Statistical Package for the Social Sciences) version 26.0 (IBM Corp., Armonk, NY, USA). Continuous variables were evaluated for normality using the Shapiro–Wilk test. Categorical variables, including gender, epistaxis recurrence, and crusting, were presented as frequencies and percentages and compared using Pearson’s chi-square test or Fisher’s exact test when expected cell counts were less than 5. Non-normally distributed continuous or ordinal variables, including age and pain scores, were expressed as medians with interquartile ranges (IQRs) and compared using the Mann–Whitney *U* test. A two-sided *P* value < 0.05 was considered statistically significant.

Participants who did not attend to receive the allocated intervention or who did not attend follow-up were not included in the final analysis. Therefore, the final analysis was performed according to a per-protocol approach rather than an intention-to-treat principle.

## Results

Of the 172 randomized participants, 83 of 86 participants in the silver nitrate group received the allocated intervention, while 3 did not attend to receive treatment. Four participants were subsequently lost to follow-up. In the TCA group, 80 of 86 participants received the allocated intervention, 6 did not attend to receive treatment, and 2 were lost to follow-up. Consequently, 79 participants in the silver nitrate group and 78 in the TCA group were included in the final per-protocol analysis. Of the 157 patients, in Group A, there were 79 patients: 36 (45.6%) females and 43 (54.4%) males, with a median age of 8 years. In Group B, there were 78 patients: 38 (48.7%) females and 40 (51.3%) males, with a median age of 7.5 years (Table 1).

**Table 1:** Comparison between the studied groups regarding demographic data

	Silver nitrate group	TCA group	*χ*^2^	*p*
	*N* = 79 (%)	*N* = 78 (%)		
Gender Female Male	36 (45.6%) 43 (54.4%)	38 (48.7%) 40 (51.3%)	0.156	0.693
	Median (IQR)	Median (IQR)	*Z*	*P*
Age (year)	8 (6–11)	7.5 (6–9)	− 1.548	0.122

*Z* Mann–Whitney test, *χ*^*2*^ chi-square test, *IQR* interquartile range

**Fig. 3:**
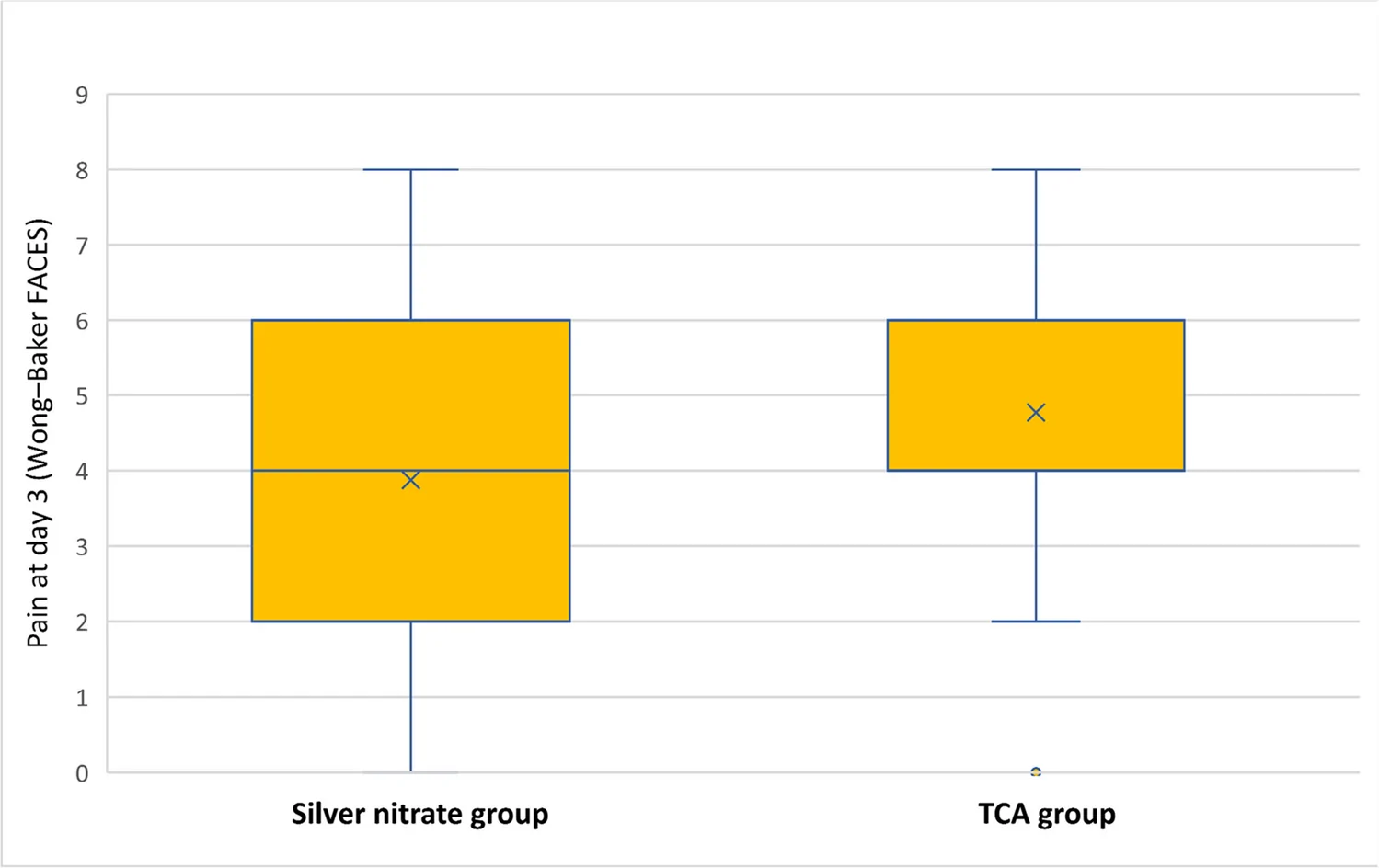
Boxplot showing a comparison between the studied groups regarding pain at third day following the procedure

There was a statistically non-significant difference between silver nitrate and TCA groups regarding gender or age while there was a statistically significant difference between the studied groups regarding pain score at day 3 following the procedure (significantly higher in TCA group) (*P* value 0.008) (Fig. 3).

Regarding recurrence of bleeding after chemical cauterization, it occurred in 15.2% of children within the silver nitrate group versus 17.9% within the TCA group; therefore, in those patients, bipolar electrical cauterization was indicated under general anesthesia to apply an electric current to the affected area to control and prevent any further attacks of bleeding.

Whereas nasal crusts occurred in 12.7% of patients within the silver nitrate group, 17.9% of patients in TCA group complained of nasal crusts. There was a statistically non-significant difference between the two groups regarding rebleeding or the presence of crust formation after 1 week following the procedure (Table 2).

**Table 2:** Comparison between the studied groups regarding outcome parameters

	Silver nitrate group	TCA group	χ^2^	*p*
	*N* = 79 (%)	*N* = 78 (%)		
Rebleeding				
No Yes	67 (84.8%) 12 (15.2%)	64 (82.1%) 14 (17.9%)	0.216	0.642
Crust at w1				
No Yes	69 (87.3%) 10 (12.7%)	64 (82.1%) 14 (17.9%)	0.848	0.357
	Median (IQR)	Median (IQR)	***Z***	***P***
Pain at day 3	4 (2–6)	5 (4–6)	− 2.645	0.008*

*Z* Mann–Whitney test, *χ*^*2*^ chi-square test, *IQR* interquartile range

*p < 0.05 is statistically significant

There were no cases complicated with septal perforation or other complications within the two groups with no irreversible discoloration detected in both study groups.

## Discussion

To our best knowledge, this is the first study to compare the efficacy of silver nitrate to TCA in epistaxis treatment. No other studies in the literature have reported such a comparison previously.

Although children’s epistaxis is usually self-limiting, it represents a bothering concern for both child and parents usually due to its idiopathic and recurrent nature. Only a small fraction of epistaxis attacks can be traced back to a known etiology like systemic coagulopathy, trauma, hereditary telangiectasia, or documented infection as vestibulitis. In more than 90% of cases, it is usually referred to as spontaneous epistaxis [10].

Classically conservative methods are otolaryngologist’s priority after the exclusion of a primary local or systemic cause. Topical nasal decongestants, nasal saline, emollient sprays, and antiseptic creams are usually first prescribed. Kubba et al. carried out a randomized controlled trial on the efficacy of antiseptic cream for recurrent epistaxis in children with a success rate of 55% [11].

In cases not responding to conservative measures, patients are often offered chemical cauterization of a visible bleeder using silver nitrate. Silver nitrate is a readily usable oxidizing agent, producing free radicals causing a hemocoagulative impact on the tissue and an antiseptic effect [12]. Calder et al. reported a statistically significant difference when using silver nitrate cautery combined with antiseptic cream in comparison to antiseptic cream only [13]. Khan et al. proved that silver nitrate cauterization was superior to anterior nasal packing, but their study was not exclusive to pediatric population [8]. On the contrary, in a double-blinded study, Ozmen et al. concluded that chemical cauterization provided better results compared to antiseptic cream, but the difference was not statistically significant [14].

Trichloroacetic acid “TCA” is a well-known cauterization agent that causes protein denaturation. It has variable uses in dermatology and ophthalmology [15, 16]. It’s also been proven safe and effective in the treatment of recurrent tracheoesophageal fistula as chemical cautery [17]. It has already been used as a treatment for inferior turbinate hypertrophy [18].

A paucity of studies shows the use of TCA in epistaxis. Sunita et al. reported the use of TCA along with silver nitrate in comparison to anterior packing in emergency epistaxis control [19]. Another study by Zhi T et al. concluded that microwave is superior to TCA and nasal packing in epistaxis [20]. Our study is the first in the literature to compare the utility of silver nitrate versus TCA in the management of anterior spontaneous epistaxis in pediatric patients through a randomized assessor-blinded trial.

Although widely used silver nitrate is in the form of readily made sticks, we have used silver nitrate solution for standardization and in order not to disturb the blinding process.

Regarding comparison outcomes between the two groups, there was no significant difference in the demographic data between the two groups. Concerning the efficacy of intervention, stoppage of bleeding was considered our only reference of success; rebleeding even one episode was accepted as a failure. Rebleeding occurred in 12 patients (15.2%) in Group A, while 14 patients (17.9%) reported recurrence in Group B. There was no significant difference between the two groups regarding recurrence. Therefore, we can consider TCA an effective alternative solution to silver nitrate.

Another point of comparison to consider is that pain score was significantly lower in the silver nitrate group. This was compatible with Khan et al. who reported significantly lower pain with silver nitrate in opposition to nasal packing [5]. Septal crusting was noticed to be slightly more in the TCA group (14, 17.9%) in comparison to silver nitrate group (10, 12.7%) which had no statistical significance.

There is a dispute concerning the optimum concentration of TCA—mainly ranging in literature from 10 to 50%—and the optimum application time used in chemical cauterization process. In our study, we used a cotton-tipped applicator dipped in 15% TCA solution applied to the bleeding point and firmly pressed for 1 min.

The lack of accurate estimation of the amount of bleeding in recurrent cases as well as the frequency of bleeding before and post intervention in failed cases represented a limitation of our study. Also, larger scale multicentric studies over long-term follow-up may be needed. Furthermore, the trial was registered retrospectively after recruitment had commenced, which should be considered when interpreting the study findings.

Also, because the pain score was reported to the study team through the parent, the possibility of reporting or recall bias cannot be completely excluded.

Therefore, regarding the clinical relevance in this study, we can consider TCA in our armamentarium as an effective chemical cautery alternative agent to silver nitrate for the management of childhood epistaxis and can be involved in commercial industrial purposes to control epistaxis in children. The use of TCA gives different opinion for the physicians when silver nitrate is not present for the epistaxis.

## Conclusion

As a treatment of idiopathic anterior epistaxis in pediatric population, both silver nitrate and TCA are easily applied, tolerable, and effective once a visible bleeder is noted, and all possible systemic and local causes of epistaxis are excluded. Although not being widely utilized in management of pediatric epistaxis, TCA is considered an effective chemical cautery agent for the management of childhood epistaxis with comparable results to silver nitrate in terms of recurrence but slightly worse in terms of pain after intervention. Although no major complications resulted in both groups, they should be utilized with caution and in suitable cases.

## Data Availability

The data sets used and/or analyzed during the current study are available from the corresponding author on reasonable request.

## Abbreviations

TCA: Trichloroacetic acid
SPSS: Statistical Package for the Social Sciences: SPSS version 26.0 (IBM Corp., Armonk, NY, USA)
